# How Couple’s Relationship Lasts Over Time? A Model for Marital Satisfaction

**DOI:** 10.1177/00332941211000651

**Published:** 2021-03-18

**Authors:** José Abreu-Afonso, Maria Meireles Ramos, Inês Queiroz-Garcia, Isabel Leal

**Affiliations:** William James Center for Research, ISPA -- Instituto Universitário, Lisbon, Portugal; William James Center for Research, ISPA – Instituto Universitário, Lisbon, Portugal

**Keywords:** Marital satisfaction, couples, predictors, structural equation modeling

## Abstract

High rates of divorce seem related to low marital satisfaction levels; however, there is still a lack of a model that can help understand the couple’s resilience and fragility throughout the life cycle. This research explores the role of communication patterns, their own and partner’s motivation for conjugality, cohesion and flexibility within a couple, and several sociodemographic characteristics (e.g., stage of the family life cycle) that can explain marital satisfaction. A sample of 331 Portuguese in a marital relationship completed a sociodemographic questionnaire and marital satisfaction measures, communication and conflict management competencies, cohesion and flexibility, and motivation. Adequate statistical analysis was performed using descriptive statistics and structural equation modeling. Both measurement and structural model performed in the study presented a good fit, with five significant predictors of marital satisfaction (that accounted for 85% of the variability): intrinsic motivation (*β* = .64), communication (*β* = .31), families with young children (*β* = −.08), families with teenagers (*β* = −.07) and professional/academic status (*β* = .06). By identifying a model for marital satisfaction, this research provides clues regarding which aspects might need to be considered in couples’ clinical work to promote healthier relationships.

## Introduction

The empirical research of marital satisfaction has shown that in stable marriages, spouses are healthier, happier, and live longer (Be et al., 2013; Robles et al., 2014; Vanassche et al., 2013; Whisman et al., 2018). Marital satisfaction refers to a global evaluation of one’s attitude towards his/her marriage, used to assess marital happiness and stability regarding all aspects of marriage (Ahmadi et al., 2010; Li & Fung, 2011; Schoen et al., 2002; Tavakol et al., 2017). Married life circumstances influence marital (dis)satisfaction (Rollins & Feldman, 1970).

The current practical advances support that marital satisfaction does not decline over time for most couples but remains relatively stable for extended periods (Karney & Bradbury, 2020). Despite exhaustive research in this area, marital satisfaction that complies with several factors – e.g., duration of the marriage, communication, attachment, conflict, and children – remains uncertain (King, 2016; Rebello et al., 2014).

During the last decade, empirical research seems to defy what most longitudinal studies of marriage consider valid. In several studies, marital satisfaction is mentioned to decrease over time (Bradbury & Karney, 2004; Pérez & Estrada, 2006), being higher in the first years of the relationship; yet, others mention that it increases again during the last stages of the life cycle (Narciso, 1994/1995, 2001; Stephen & John Michael Raj, 2014). Some couples mention decreased marital satisfaction based on a retrospective evaluation, although acknowledging recent improvements counterbalance that decrease (Karney & Frye, 2002). Also, older couples might present higher marital satisfaction levels since unsatisfactory unions end in divorce (Henry et al., 2007).

Studies show that most marriages end in divorce (Schoen & Canudas-Romo, 2006), mainly due to low levels of satisfaction—with Portugal presenting a divorce rate of 58,7%, the second-highest in Europe (Fundação Francisco Manuel dos Santos, 2020). Researchers remain lean towards a better understanding of which factors contribute to higher satisfaction, which will allow marital counselling and couples to employ strategies that may contribute to a more satisfying marriage.

### Marital satisfaction over the family life cycle

The family life cycle shows different stages of family members' developmental processes and related risks (Duvall, 1967; Neighbour, 1985; Relvas, 2004). They are considered a framework for analyzing marital success (including variables as satisfaction, happiness, or social expectations; Burgess & Locke, 1945).

The significant changes in family undergo over time as an important factor that affects and impacts marital satisfaction. Family therapists realized that it is essential to contextualize couples' crisis in the family life cycle, claiming that transition phases might reflect higher vulnerability than others (Haley, 1984). Waldemar (2008) approaches these with newly formed couples and highlights the need to deal with a series of questions, as the bonding process with the family of origin or the partner's idealization. If the couple succeeds in managing these, then the birth of a first child leads to another complex transition. This transition can be seen both ways: negative – the couple experience exhaustion, lack of time for themselves, and more disagreement – and in a positive one – a sense of gratification and joy (e.g., Belsky & Pensky, 1988; Cowan & Cowan, 1993; Twenge et al., 2003). This period needs special attention since it introduces additional stress to the couple relationship, accelerating the decline in marital satisfaction (e.g., Belsky & Pensky, 1988). In this stage, the romantic relationship is usually relegated to second place, conflicts concerning children’s education might occur, paradoxical social pressures are felt, and the first sexual difficulties might emerge. If disputes between partners remain unresolved, they may increase when children reach adolescence. This new stage is considered one of the critical periods for the marital relationship at midlife (e.g., Steinberg & Silverberg, 1987). This period will coexist with the couple’s existential crisis, with each partner probably 40–50 years old, reassessing their lives and redefining its meaning while also dealing with their parents’ aging. Another essential stage concerns children’s departure and the re-adaptation of living together as a couple; this stage can concurrently occur with the couple trying to manage their parents’ death and retirement (Waldemar, 2008). When couples experience an improvement in their relationship as they age and children leave home, the decline in satisfaction with their relationship eventually reverses (Gorchoff et al., 2008). Couples with children remain at home after reaching adulthood (Umberson et al., 2005), which might become a problematical paradoxical situation. Finally, another nodal point of the couple’s life is the aging of the dyad and the closeness to death (Waldemar, 2008).

### Predictors for marital satisfaction

According to Narciso and Costa (1996), the relationship’s quality and marital satisfaction in different areas of the couple’s life are concerned throughout two crucial dimensions: love and conjugal functioning. Conjugal functioning refers to how a couple organizes and manages relationships within their conjugal/family holon, covering aspects such as roles and functions, free time, autonomy/privacy, communication and conflicts, and extra-family relationships. Love is related to feelings that each member of the couple has for each other or their relationship. Love would then cover aspects such as feelings and emotional expression, sexuality, emotional intimacy, the sense of continuity of the relationship, and the opinion on the partner’s physical and psychological characteristics (Narciso & Costa, 1996). How couples manage their differences and problems during their life cycle might be a relevant factor to distinguish between satisfied and unsatisfied couples (Hall, 2006; Markman, 1992), which leads us to consider the impact of significant components on relationships’ success (i.e., communication, marital beliefs, the family of origin, idealization of the partner).

Several authors acknowledge communication as strongly associated with marital satisfaction and stability (Alayi et al., 2011; Haris & Kumar, 2018; Lavner et al., 2016; Rehman & Holtzworth-Munroe, 2007; Shafer et al., 2014). Positive and negative communication competencies are good predictors of marital satisfaction (Johnson et al., 2005). Generally, satisfied couples reveal more constructive communication patterns—showing positive communication behaviors and seeking to avoid negative ones—while more problematic couples predominantly adopt destructive styles of communication—frequently calling upon negative styles of problem’s resolution strategies, mostly related to high levels of offense and unresolved issues (Bertoni & Bodenmann, 2010; Birchler & Webb, 1977; Bradbury & Fincham, 1992; Houts et al., 2008). Consequently, couples with unsolved problems that may be under distress may be at risk of increased harmful and destructive communication patterns challenging to resolve, resulting in decreased couple’s intimacy (Eldridge et al., 2007; Pearce & Halford, 2008). Contrary to couples that can engage in joint and shared effort solving, leading to higher levels of the couple’s satisfaction (South et al., 2010).

The quality of marital relationships is not static, with communication facilitating marital dynamics modifications (Olson & Gorall, 2003). Couples’ cohesion and flexibility throughout life also seem essential to couples’ satisfaction and significant meaning during family transitions (Olson, 2000). Cohesion, varying between separateness and togetherness, can be defined by the emotional bonding that family members and couples have towards two crucial dimensions. Flexibility, ranging between stability and change, can be characterized by the number of leadership changes, roles, and rules within the relationship (Olson, 2000; Olson & Gorall, 2003). Moderate levels of cohesion and flexibility allow couples and families to balance between separateness and togetherness and between stability and change according to the situation lived, resulting in more functional systems (Olson, 2000; Olson & Gorall, 2003). However, this does not mean that couples and families will never adopt behaviors that are characteristic of extreme levels to deal with certain situations (Barnes & Olson, 1985; Olson & Gorall, 2003). Excessive levels of cohesion and flexibility can be useful in some cases but generally be pejorative if the marital and family functioning remains in those (Olson, 2000). Poor communication within a couple does not lead to changes in the marriage functioning, which results in families maintaining themselves in extreme levels of cohesion and flexibility (Olson, 2000; Olson & Gorall, 2003).

Another factor that seems vital to predict marital satisfaction in the relationship is both partners and their motivational styles (Blais et al., 1990). Motivation influences the establishment and maintenance of relationships, the choice of the partner, the quality of everyday relational behaviors, and the development and breakdown of relationships (Aimé et al., 2000; Blais et al., 1990; Deci & Ryan, 2000, 2008; Knee et al., 2002; Patrick et al., 2007; Rempel et al., 1985). Notably, it seems more likely that a relationship will last and be more satisfactory if the motivation is more intrinsic than extrinsic (Rempel et al., 1985), with several authors claiming that more adaptive behaviors (e.g., when dealing with conflicts) emerge if the motivation for being in a relationship is intrinsic and autonomous (Blais et al., 1990; Knee et al., 2002; Patrick et al., 2007). Extrinsic motivation might weaken intrinsic motives and reduce the feeling of love for the partner (Rempel et al., 1985; Seligman et al., 1980). Additionally, faith in a relationship also seems to be associated with the perception that the partner is intrinsically motivated (Rempel et al., 1985). Likewise, couples who present congruent and self-determined motivational styles appear to have high marital satisfaction levels (Aimé et al., 2000).

Dyadic coping also seems a crucial element to predict marital satisfaction. Current empirical studies have stated that dyadic coping can help stress communication and manage individual strategies to cope with stressful situations (Falconier et al., 2015). Research inclines to more prevention and intervention for all couples, implementing dyadic coping as a behavioral skill to help couples in unsatisfied relationships (Falconier et al., 2016).

Research on marital satisfaction has also suggested that differences between sexes might be essential in explaining a couple’s satisfaction and adjustment. For example, differences between men and women in expressing and dealing with emotions (Cordova et al., 2005; Mirgain & Cordova, 2007; Rauer & Volling, 2005), managing conflicts and problems (Miller et al., 2003) can influence how partners communicate with each other, thus helping to understand couples’ dynamics. Nonetheless, some authors, such as Dandurand and Lafontaine (2013), state no significant differences between men and women regarding intimacy and marital satisfaction. Findings reinforce more future interpolations acknowledging sociodemographic predictors for marital satisfaction. Significant correlations were found within sex, communication skills, duration of the marriage, conflict resolution styles, attachment styles, and educational level (Kardan-Souraki et al., 2018; Zainah et al., 2012; Zhang et al., 2016).

### The present study

More evidence of what maintains a healthy marriage and higher marital satisfaction is needed to help couples in crisis. This is crucial for maintaining long-term relationships since the family goes through several life moments that require adaptation and changes. Although changes are a way of dealing with a crisis, couples can encounter an unstable moment, increasing dissatisfaction and stress (Olson, 2000). Few studies explore the stability and growth in the family life cycle, especially at intermediate stages. Most of them give little information about what problems couples face at different stages (Miller et al., 2003).

Our primary interest came from understanding significant marital satisfaction predictors that have important implications in a clinical context. First, we wanted to understand what happens along the life cycle of satisfied couples and what makes them stay together most of their lifetime. Second, helping couples in crisis requires knowledge of what keeps a marriage healthy so that clinicians may be better able to predict which couples are at higher risk of divorce. In the absence of explanatory models of the dynamics of the family life cycle, our study aims to understand the factors that work as promoters of the couple’s resilience, allowing them to maintain the relational quality and the fragility factors of considerable risk. To our understanding, there is the lack of a model based on a set of concepts and psychological variables that, by influencing marital quality and stability, may be crucial to the success of relationships, and that according to literature and following our clinical experience are essential to assess satisfaction within a couple. Most early studies focused on cross-sectional designs, limiting information about how marriage unfolds over time (Hirschberger et al., 2009). Spite the growing longitudinal studies of marriage, many suffer from methodological problems (e.g., such as not covering part of the duration of a marriage or not measuring marital satisfaction throughout the study).

To develop the model, it was considered how marital satisfaction might be explained by communication, motivation, cohesion, flexibility, stages of the family life cycle, and sex, alongside with other sociodemographic variables (e.g., age, professional/academic status, educational level, union type, union duration, and current union being the first person’s marriage/cohabitation or not).

## Method

### Participants

The present study is part of a larger project examining different aspects of the couple’s dynamics during life, for which 596 valid research protocols were collected. However, considering the study’s aim, the final sample was formed by 331 heterosexual participants, 72.8% married, and 27.2% living in cohabitation. The ages’ oscillated from 19 to 80 years old; more than 50% of the sample had higher education and was professionally active. Two hundred twenty-nine married participants have children (95%), and 49 participants living in cohabitation also have children (54,4%). Table 1 summarizes the characteristics of the sample.

**Table 1.** Characterization of participants (N = 331) in relation to sociodemographic variables.

	*n*	**%**
Sex		
Male	165	49.8
Female	166	50.2
Age (*M*; *SD*)	42.54 (12.30)	
<30	47	14.2
30–39	106	32.0
40–49	83	25.1
50–59	57	17.2
60–69	33	10.0
>70	5	1.5
Professional/academic status		
Active (employed, student, student-worker)	269	81.3
Inactive (unemployed, retired)	51	15.4
No answer	11	3.3
Educational level		
Without higher education	185	55.8
With higher education	144	43.4
No answer	2	.6
Family life cycle stage		
Beginning families	57	17.2
Families with young children	76	23.0
Families with school-age children	48	14.5
Families with teenagers	65	19.6
Families whose children have left home	15	4.5
Families with adult children staying at home	43	13.0
Families in the middle years	27	8.2
Union type		
Marriage	241	72.8
Cohabitation	90	27.2
Union duration (*M*; *SD*)	16.40 (12.32)	
<5 years	68	20.5
5–9 years	60	18.1
10–14 years	42	12.7
15–19 years	34	10.2
20–29 years	69	20.9
≥30 years	58	17.5
First marriage/cohabitation		
Yes	290	87.6
No	41	12.4
Presence of childrenª		
Yes	278	84.0
No	53	16.0
Number of childrenª		
0	53	16.0
1	92	27.8
2	136	41.1
3	40	12.1
>3	10	3.0

*Note. M* = mean; *SD* = standard deviation.

ªCurrent and/or previous relationships.

Inclusion criteria were: (a) individuals who were married to or cohabiting with the partner and (b) according to the literature, participants could be integrated as part of only one of the family life cycle stages (Neighbour, 1985; Relvas, 2004; Umberson et al., 2005; Waldemar, 2008). However, a new group was considered. We came across several couples that still had their adult children living with them. This raised a question about the couple’s effect, which is expected to be in a different stage and a common situation in our culture today. Umberson et al. (2005) study acknowledged that adult children’s presence causes tensions between the couple, instigating low levels of positive marital experiences. As such, it was considered the following criteria regarding the distribution of the sample across groups:

1. *Beginning families*, participants that were married or living together up to four years (inclusive) without children from current and/or previous relationships residing with them. All participants with more than five years of marriage/cohabitation and less than four years of marriage/cohabitation with children were excluded.
2. *Families with young children*, participants with children from the current relationship with age up to five years old, regardless of the number of years of marriage/cohabitation. Given the importance of assessing the impact of the child’s birth, participants who had children from previous relationships were excluded.
3. *Families with school-age children*, participants with children aged between six and 12 years old (inclusive), regardless of years of marriage/cohabitation. Participants with children at these ages who also had older children (from current and/or previous relationships living with them) were excluded.
4. *Families with teenagers*, participants with children aged between 13 and 19 years old. Participants with children at these ages but that also had older children (from current and/or previous relationships) living with them were excluded.
5. *Families whose children have already left home*, participants whose children left home for at least four years (inclusive). Although have some children have left home, it was excluded participants still have others living with them.
6. *Families with adult children staying at home*, participants with adult children (aged over 23 years) still live at home. Participants whose children were aged between 20 and 23 (at university attendance cannot be considered adults or teenagers) were excluded.
7. *Families in the middle years*, participants without children at home and with at least one member of the couple aged 60 years old. All participants were included regardless of the number of marriages/cohabitations and children from current and/or previous relationships.

### Measures

A sociodemographic questionnaire was prepared by the authors of this research for the sample’s characterization and to define the groups of this study. All participants were asked to provide their sex, age, professional/academic status, educational level, couple’s information (e.g., type [marriage or cohabitation] and duration [in years] of the marital union), and the number of children (current and/or previous relationships), their ages and situation (still living at home or already left home). Participants also completed marital satisfaction measures, communication and conflict management competencies, cohesion and flexibility, and motivation.

#### Marital satisfaction

A self-report questionnaire that assesses satisfaction within a couple’s relationship using the Scale of Evaluation of the Satisfaction on the Marital Areas of Life (EASAVIC; Narciso & Costa, 1996), through a total of 44 items, in a 6-point Likert scale from *not satisfied at all* to *completely satisfied*. These items represent different areas of the couple’s life that could be considered in two dimensions: (1) Functioning that comprise areas such as familiar functioning (roles and functions, autonomy/privacy), free time, communication and conflicts, and relationships outside of the family; (2) and Love, with areas such as feelings and emotional expression, sexuality, emotional intimacy, continuity of the relationship, and physical and psychological traits. A global average score includes the two dimensions. The EASAVIC presents good reliability values, revealing Cronbach’s alphas higher than .90 (Narciso & Costa, 1996).

#### Styles and patterns of communication

Specific competencies of communication and conflict management were evaluated through the Managing Affect and Differences Scale (MADS; Arellano & Markman, 1995; Portuguese version by Abreu-Afonso & Leal, 2016a). The self-report measure comprises 109 items, and a 5-point Likert scale from *strongly disagree* to *agree strongly*. The Portuguese version of the MADS is organized in dimensions that reflect either constructive or destructive communication patterns: (1) Emotional Expressiveness and Positive Communication, which relates to love, affection and the degree of comfort with emotional expression; (2) Negativity/Negative Escalation, which regards the expression of negative attitudes and feelings; (3) Clarification, which relates to asking the partner how he/she is feeling and talking about his feelings; (4) Availability and Affective Expression, which involves love and affection for the partner, the availability to listen to him/her and to share own emotions; (5) Focusing/Stop Actions, which reflects behaviors as talking about an issue at a time or stop an escalating conflict agreeing to postpone the discussion for a more adequate moment; (6) Editing/Validation, which represents controlling one’s reactions to a partner’s message and to expressing value in partner’s perspective or point of view; (7) Withdrawal, which involves physically or emotionally withdrawing from discussions; (8) Feedback, which consists in paraphrasing or asking clarifications of partner’s message; and (9) Communication Over Time, which involves improvements in communication over time within a couple. In the Portuguese version (Abreu-Afonso & Leal, 2016a), good reliability levels were found for all the nine dimensions, with Cronbach’s alphas varying between .60 and .93.

#### Cohesion and flexibility

Couples’ dynamics were measured through the couple’s version of the Family Adaptability and Cohesion Evaluation Scale III (FACES III; Olson et al., 1985; Portuguese version by Abreu-Afonso & Leal, 2016b) in a 5-point Likert scale from *almost never* to *almost always*. This scale is composed of 20 items and divided into two dimensions: (1) Cohesion (10 items), which assesses emotional bond, support, family limits, free time and friends, recreative interests and activities; and (2) Flexibility (10 items), which assesses leadership and control, negotiation, roles, and rules. The Portuguese version of the FACES III revealed good reliability levels, with Cronbach’s alphas varying between .70 to .89 (Abreu-Afonso & Leal, 2016b).

#### Motivation

The motivation assessment was made using the Motivation Scale (MS; Rempel et al., 1985; Portuguese version by Abreu-Afonso & Leal, 2009). This scale has 24 items answered on a 9-point Likert scale from *nothing* to *completely* assess both Intrinsic Motivation and Extrinsic Motivation. Participants should answer the MS twice, considering two different perspectives: firstly, they should answer regarding their viewpoint, which therefore allows assessing their motivation to conjugality; secondly, they should answer regarding their view of the spouse/partner’s reasons to be in the relationship, which therefore allows assessing perceived motivation. MS, thus, allows setting four dimensions for each participant: (1) Intrinsic Personal Motivation, (2) Extrinsic Personal Motivation, (3) Intrinsic Perceived Motivation, and (4) Extrinsic Perceived Motivation. In the Portuguese version (Abreu-Afonso & Leal, 2009), good reliability levels were found, with Cronbach’s alphas varying between .87 and .96.

### Procedure

The Ethics Committee approved the study of ISPA – Instituto Universitário and the institutions where the sample was recruited before data collection. Also, the study was under the ethical Declaration of Helsinki and later amendments. Written informed consent was presented to the participants clarifying the study’s purpose, the research’ collaboration procedure, the confidentiality, and anonymity of the information collected. All potential participants were informed that it was voluntary participation, that they could skip any question that they did not answer, and that they could withdrawal at any moment without consequences. The research protocol was distributed and delivered to easy access points as several private and public services in the Lisbon metropolitan area (e.g., schools, businesses, health centers, community centers). Individuals were recruited through a “snowball” sampling system for a total of 18 months and received no financial incentives.

### Data analysis

All analyses were done with the IBM SPSS Statistics and AMOS statistical software, both version 25. Regarding preliminary analysis, the data normality was assessed, and missing values were imputed for variables through the mean interpolation method, where its frequency was lower than 5% of the sample. Descriptive statistics for marital satisfaction, communication, motivation, cohesion and flexibility, and sociodemographic variables were performed.

The quantification of love and functioning, styles, and patterns of communication, own’s and partner’s type of motivation, cohesion, and flexibility within the couple’s dynamics and sociodemographic variables were integrated into a structural equation model to assess marital satisfaction. Only total scores of each construct (i.e., subscales) of the instruments used were considered as observed variables for this analysis. Additionally, to integrate stages of the family life cycle in this model, each stage was transformed into a dichotomous variable (“yes/no”). Also, the sociodemographic variables were transformed into a dichotomous variable (sex: male/female; professional/academic status: inactive/active; educational level: without education/with education; union type: marriage/cohabitation; first marriage/cohabitation: yes/no).

Multicollinearity between independent variables was explored with the variance inflation factor (VIF); almost all variables presented VIF values below 5, revealing the absence of collinearity (Hair et al., 2014). The only two exceptions were for: (1) age, new couples, couples with young children, and union duration, which led us to remove age and union duration from the analyses since they presented higher VIF values and regarding our interest in studying the impact of stages of the family life cycle on marital satisfaction; (2) Intrinsic Personal Motivation and Intrinsic Perceived Motivation; however, since they are part of the same construct (i.e., intrinsic motivation) and are assessed with the same set of items—yet asking participants to take their perspective (for Intrinsic Personal Motivation) firstly and then to take their partner’s perspective (for Intrinsic Perceived Motivation)—we did not consider unreasonable to take both into account to perform our analyses.

Therefore, the structural equation model for marital satisfaction was built relating it with 13 independent variables: sex; professional/academic status; educational level; union type; first marriage/cohabitation; beginning families; families with young children; families with school-age children; families with teenagers; communication; intrinsic motivation; extrinsic motivation; cohesion and flexibility. Two stages of the family life cycle—families whose children have left home (*n* = 15) and families in the middle years (*n* = 27)—were not included in the model due to fewer participants. The family life cycle stage with adult children staying at home was also not included in the model due to not revealing a normal distribution according to skewness values (Sk > 3; Kline, 2015). The goodness of fit of the model was given by chi-squared statistics (χ^2^/df), comparative fit index (CFI), goodness of fit index (GFI), and root mean square error of approximation (RMSEA). Reference values used to evaluate the goodness of fit were practiced in structural equation modeling (Byrne, 2016).

A two-step approach was used to evaluate the structural model. Firstly, the factor’s measurement model was assessed to demonstrate an acceptable fit. Observed variables with standardized regression weights inferior to .40 were removed if their removal did not compromise the theoretical meaning of the model and correlations between variables that were not significant; errors of measurement were also progressively correlated based on modification indices. Secondly, the structural model encompassing the dependent and the 13 independent variables was built, and the significances of the structural trajectories were assessed.

## Results

Descriptive statistics regarding both mean and standard deviation values for marital satisfaction, communication, cohesion and flexibility, and motivation are presented in Table 2.

**Table 2.** Mean and standard-deviation of marital satisfaction, communication, cohesion and flexibility, and motivation variables.

	*M*	*SD*	Scale range
EASAVIC			
Marital satisfaction	4.41	.79	1–6
Love	4.56	.85	1–6
Functioning	4.19	.77	1–6
MADS			
Emotional expressiveness/positive communication	3.70	.55	1–5
Negativity/negative escalation	2.65	.67	1–5
Clarification	3.86	.52	1–5
Availability and affective expression	4.21	.50	1–5
Focusing/stop actions	3.19	.67	1–5
Editing/validation	3.69	.56	1–5
Withdrawal	2.59	.84	1–5
Feedback	3.59	.67	1–5
Communication over time	3.60	.80	1–5
FACES III			
Cohesion	40.57	6.35	10–50
Flexibility	33.59	5.99	10–50
MS			
Intrinsic personal motivation	6.63	1.37	1–9
Extrinsic personal motivation	3.93	1.66	1–9
Intrinsic perceived motivation	6.52	1.35	1–9
Extrinsic perceived motivation	4.06	1.67	1–9

*Note. M* = mean; *SD* = standard deviation.

Alongside with high satisfaction levels among participants—both considering Marital Satisfaction and its dimensions, Love and Functioning— dimensions reflecting constructive communication patterns presented higher mean values than dimensions reflecting destructive communication patterns. Additionally, according to cut-off values previously established by Abreu-Afonso and Leal (2016b), mean values for both Cohesion and Flexibility reflected moderate levels. Regarding motivation, this sample showed higher mean values for Intrinsic Motivation dimensions than for Extrinsic Motivation ones.

The fit of both the measurement and structural models (Table 3) was good, and some correlations between measurement errors were performed. It should be noted that the observed variables Focusing/Stop Actions and Communication Over Time were removed due to their standardized regression weights on Communication being lower than .40, and since we did not consider that those removals would compromise the theoretical meaning of the model.

**Table 3.** Model goodness fit indexes for factor anal*ysis.*

	χ^2^/df	CFI	GFI	RMSEA
Measurement model	2.085	.944	.907	.057
Structural model	2.038	.973	.947	.056

Bivariate associations between independent and dependent variables are presented in Table 4, and standardized structural weights of the independent variables regarding marital satisfaction are shown in Table 5.

**Table 4.** Bivariate associations for the independent and dependent variables.

	S	PAS	EL	UT	FMC	BF	YC	SAC	T	C	IM	EM	CF	MS
S	1													
PAS	−.01	1												
EL	.03	.22***	1											
UT	.01	.22***	.22***	1										
FMC	−.10	.03	.11*	.35***	1									
BF	.01	.17**	.15**	.50***	.17**	1								
YC	−.00	.16**	.07	.15**	.04	−.25***	1							
SAC	.02	.14**	−.02	−.10	.00	−.19***	−.23***	1						
T	−.01	.05	.01	−.23***	−.12*	−.23***	−.27***	−.20***	1					
C	−.04	.13*	.06	.22***	.06	.30***	−.03	.05	−.11	1				
IM	−.02	.10	.02	.15*	.02	.21***	.02	.06	−.12	.84***	1			
EM	−.21***	−.12*	−.25***	−.09	−.04	−.00	−.10	.03	.05	.22***	.42***	1		
CF	.07	.09	.02	.18**	.06	.22***	−.03	.04	−.09	.82***	.86***	.21**	1	
MS	−.04	.15**	.02	.17**	.00	.26***	−.04	.07	−.14*	.86***	.89***	.28***	.84***	1

*Note.* S = Sex; PAS = Professional/Academic Status; EL = Educational Level; UT = Union Type; FMC = First Marriage/Cohabitation; BF = Beginning Families; YC = Families with Young Children; SAC = Families with School Age Children; T = Families with Teenagers; C = Communication; IM = Intrinsic Motivation; EM = Extrinsic Motivation; CF = Cohesion and Flexibility; MS = Marital Satisfaction.

*p ≤ .05; **p ≤ .01; ***p ≤ .001.

**Table 5.** Standardized structural weights of the independent variables (sociodemographic, motivation, cohesion and flexibility and communication variables) regarding the dependent variable (marital satisfaction).

Trajectories			*β(SE)p*
Marital satisfaction	←	First marriage/cohabitation	−.04(.06).198
Marital satisfaction	←	Union type	.00(.06).996
Marital satisfaction	←	Professional/academic status	.08(.06)**
Marital satisfaction	←	Educational level	−.02(.04).405
Marital satisfaction	←	Sex	−.04(.04).130
Marital satisfaction	←	Families with teenagers	−.09(.06)**
Marital satisfaction	←	Families with school-aged children	−.03(.07).451
Marital satisfaction	←	Families with young children	−.10(.06)**
Marital satisfaction	←	Beginning families	−.03(.08).544
Marital satisfaction	←	Intrinsic motivation	.56(.05)***
Marital satisfaction	←	Extrinsic motivation	−.04(.02).236
Marital satisfaction	←	Cohesion and flexibility	.16(.01).057
Marital satisfaction	←	Communication	.26(.09)***

Note. *β* = standardized estimates; *SE* = standard error; *p* = significance level.

**p ≤ .01; ***p ≤ .001.

**Figure 1.**
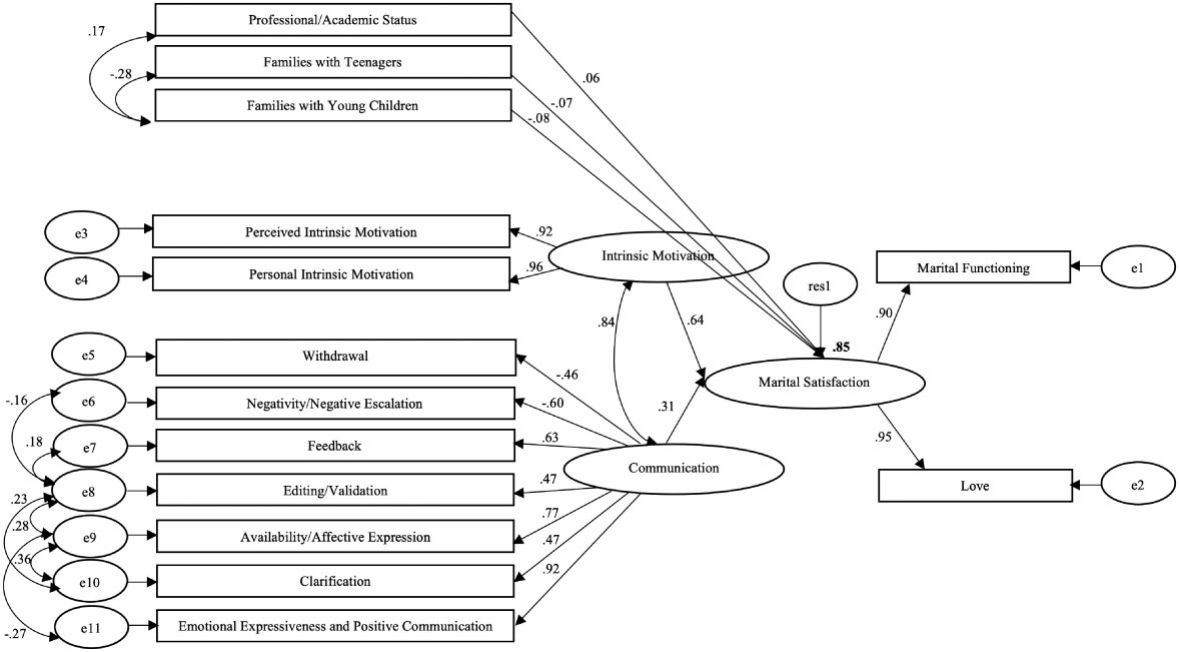
Structural model for marital satisfaction: Its relation with communication, intrinsic motivation and significant sociodemographic variables. One direction arrows represent significant trajectories and two direction arrows represent significant correlations, p ≤ .05.

Five variables in a total of 13 are significant predictors of Marital Satisfaction, accounting for 85% of Marital Satisfaction variability. Intrinsic Motivation reveals the highest standardized structural weight in explaining Marital Satisfaction, followed by Communication. In contrast, Intrinsic Motivation is positively explained by personal Intrinsic Motivation and by perceived Intrinsic Motivation, and Communication is positively explained by constructive communication patterns and negatively explained by destructive communication patterns. Additionally, it is suggested that Marital Satisfaction is better explained as participants do not have young children, do not have teenagers, and are professionally and/or academically active. The structural model, with only its significant trajectories and correlations, is shown in Figure 1.

## Discussion

This study’s central aim was to explain marital satisfaction by integrating and analyzing in a structural model several variables that have long been suggested as important to marital quality and stability and, consequently, for relationships’ success. Given the high divorce rates, it seems imperative that we understand the critical risks to marital quality and stability.

Our results showed that the structural model obtained presented a good fit and explained most of the variance of marital satisfaction—thus contributing both to literature growth given the lack of a model that embraces the variables studied, and to family and couple therapists’ knowledge on which features might be necessary for maintaining couples together and in promoting healthier relationships.

Regarding motivation, while Extrinsic Motivation did not significantly explain marital satisfaction, Intrinsic Motivation was revealed to be the strongest predictor in the structural model. This result is in line with the literature, stating that intrinsic motives better account for a relationship’s satisfaction and success (Rempel et al., 1985; Seligman et al., 1980) and people’s happiness (Sheldon et al., 2004) compared to extrinsic motives. Interestingly, this study revealed Intrinsic Motivation and Communication to be strongly associated in explaining marital satisfaction, which again supports existing literature stating that self-determined motivation styles seem to reflect more adaptive behaviors, inclusively in conflict resolution (Blais et al., 1990; Knee et al., 2002; Patrick et al., 2007), which recalls for constructive communication patterns. In general, communication was also pointed as a strong predictor of marital satisfaction, and its relation with satisfaction within a couple was positively explained by emotional expressiveness and positive communication, clarification, availability and affective expression, and editing/validation. On the other hand, it was negatively explained by negativity/negative escalation and withdrawal. This had also been acknowledged by other authors, stating that both positive and negative communication behaviors predict marital satisfaction (Johnson et al., 2005) and that constructive communication patterns are more characteristic of satisfied couples (Bertoni & Bodenmann, 2010; Birchler & Webb, 1977; Bradbury & Fincham, 1992; Houts et al., 2008), whereas negative interactions seem positively correlated with thinking about divorce (Stanley et al., 2002). Observational studies of couple interaction refer that improved communication, once achieved, will enhance the quality and stability of the relationship (Karney & Bradbury, 2020).

Generally, moderate levels of Cohesion and Flexibility are associated with more functional family and couple’s systems (Olson, 2000; Olson & Gorall, 2003). Nevertheless, despite moderate mean values found among our sample, Cohesion and Flexibility were not significant in explaining marital satisfaction in the structural model. This outcome might be because functional families and couples, notwithstanding usually organizing themselves within moderate levels of Cohesion and Flexibility, may engage in behaviors characteristic of extreme levels in the face of particular situations (Barnes & Olson, 1985; Olson & Gorall, 2003). This subjectivity was not assessed in this study, and we suggest that cohesion and flexibility might be more informative regarding marital satisfaction if assessed in a clinical context, asking couples to answer twice to FACES III—first concerning how they perceive their marital relationship (perceived couple) and then how they would desire their marital relationship to be (idealized couple; Abreu-Afonso & Leal, 2016b; Olson et al., 1985). Answers regarding the idealized couple would suggest each subject’s preferences and guide clinical work towards eventual changes to pursue in the marital relationship (Maynard & Olson, 1987).

Concerning the structural model found, there also seems to be a tendency for less satisfaction when couples have young children, and when they have teenagers instead of being in other stages of the family life cycle. Indeed, the transition to parenthood is stated as bringing several changes to the couple’s dynamics, consequently resulting in increased stress and decreased positive aspects of the relationship (e.g., sex, affection; Lavner et al., 2014; Waldemar, 2008). Additionally, during children’s adolescence, there is a change in parental roles to allow teenagers to move in and out of the family system, which might happen along with conflicts within the couple regarding the end of the reproductive life, career issues, and their higher parents’ dependence derived from the aging process. Despite a few studies on marital satisfaction when parenting teenagers, Pérez and Estrada (2006) stated higher couple’s intimacy until children reach adolescence, and Cui and Donnellan (2009) suggested that conflict between a parent and a child in this stage may also result in disputes between spouses, having an impact on couple’s relationship. These authors found a decline in marital satisfaction during children’s adolescence (Cui & Donnellan, 2009).

According to our findings, marital satisfaction also seems to be better explained by being professionally and/or academically active than being unemployed or retired. For example, Howe et al. (2004) had studied job loss in couples. These authors had achieved an integrated view on the issue, stating that both the unemployed subject and his/her partner might feel distressed after a job loss, mutually reinforcing the distress felt by the other member of the couple and, consequently, relationships’ quality can become compromised—which is in line with our results. It is also noteworthy that marital satisfaction seems higher within couples with congruent attitudes concerning their providers’ roles and fairer and more equal division of the housework (Helms et al., 2010). While men and women traditionally had different but complementary roles, nowadays, society brings new challenges to the couple due to higher gender equality, more valued individuality (Aboim, 2006), and family structure changes. According to our structural model, this fact may also explain the non-significance of the subject’s sex on marital satisfaction. There is a lack of consensus in the literature on differences between sexes and how this might impact the couple’s relationship. Our results support Dandurand and Lafontaine (2013) conclusions stating no differences in marital satisfaction between men and women. Nonetheless, future research should also address this issue to clarify incongruent findings across existing studies.

Finally, marital satisfaction did not reveal to be explained by subjects’ educational level, union type, and current union being the first person’s marriage/cohabitation. According to the literature, cohabitation is usually associated with reduced levels of satisfaction and happiness compared to marriage (Vanassche et al., 2013), and there seem to be contradictory findings regarding marital satisfaction differences between marriages and re-marriages (Mirecki et al., 2013). To our knowledge, not much literature exists on the eventual influence of education on marital satisfaction. Future studies are then needed regarding these variables and results.

Some limitations of this research must, although, be considered. For clinical reasons, we aimed to understand what made satisfied couples work. However, this decision may have limited a global understanding of marital realities, namely on potential other features of unsatisfied couples’ relationships, not directly addressed in this study. Thus, it would be pertinent to reproduce this research with couples with low satisfaction levels and in therapy, eventually also accounting for potential changes in the variables considered in this study throughout the therapeutic process. Since we only considered Portuguese and heterosexual subjects, it can also be relevant to include samples from other nationalities and sexual orientations in future studies to assess the current results’ generalization.

More research is also needed concerning marital satisfaction and stages of the family life cycle. Indeed, couples whose children have left home, in old age, and with adult children staying at home were not directly accounted for in the present study on their potential role in explaining marital satisfaction. This limitation was due to either a reduced number of subjects or a skewed data distribution, which calls for more representative samples regarding each stage of the family life cycle in further research. Moreover, couples with adult children staying at home represent an emergent stage of the family life cycle, and its impact on marital relationships is not yet known, which must be clarified. It should also be noted that this research was cross-sectional; other studies should adopt a longitudinal design to assess the generalization and stability of the predictors of marital satisfaction found.

Finally, the use of self-report measures entails difficulties in ensuring both subjects’ full understating and sincerity when answering the instruments’ items, making it difficult to control answers’ reliability. Therefore, the present results would benefit from being interpreted with complementary information obtained with other methodologies—for example, qualitative methods. Our structural model’s variables explained 85% of marital satisfaction variability, although other unexplored variables in this study should be analyzed further. For instance, neither age nor union duration was explored as predictors of marital satisfaction since multicollinearity was found between these two variables and two stages of the family life cycle. We hypothesize, though, that multicollinearity was revealed because as people age and spend more years together, it can also be expected that they will experience new stages of the family life cycle.

Couples experience challenges and difficulties inherent to events of the life cycle, whether they are satisfied with their marital relationship or not. Therefore, investment in both basic and applied research seems essential, resulting in an increased understanding of how relationships work and how they will develop and maximize clinicians’ and therapists’ therapeutic efficiency approaches to couples. Accordingly, and despite the limitations discussed, we believe that the present research provides important clues regarding what might need to be endorsed in therapy to avoid the relationship’s dissolution when facing a crisis. Specifically, by acknowledging which features seem to increase marital satisfaction, we believe that a path is revealed not merely to increase marital bond and commitment, but more importantly, couples’ and families’ quality of life.

## Notes

**Author Biographies:** **José Abreu-Afonso, **PhD, is a psychoanalyst of Sociedade Portuguesa de Psicanálise (S.P.P.) and International Psychoanalytic Association (I.P.A.). He is also the vice-president of S.P.P. Professor and researcher at ISPA – Instituto Universitário, in Lisbon. He is the pedagogical coordinator of the internships in Clinical and Health Psychology Master degree. His clinical experience included institutional work, and he is currently developing in private practice with adults, adolescents and families. In addition, he is a supervisor of psychotherapists in independent and institutional practices. Author of several works published in scientific journals, books and abstracts of national and international events. **Maria Meireles Ramos,** MSc in Clinical and Health Psychology, was a research assistant in William James Center for Research, ISPA – Instituto Universitário at the time of this study. As research assistant, she collaborated in diverse research projects on the translational line and has co-authored several publications. Since 2019, she has been working as a Psychologist in schools, intervening essentially with children, adolescents, families and teachers. **Inês Queiroz-Garcia, **MSc in Clinical Psychology, since 2018, has collaborated on several (inter)national projects in a research assistant position at the William James Center for Research (R&D), ISPA – Instituto Universitário. The latest collaboration was as a research assistant of an R&D project funded by a BIAL Foundation Grant (ref. 188/18) – “COping with PAin through Hypnosis, mindfulness and Spirituality”, focusing on the effects of three psychosocial interventions experimentally induced pain. Inês is a PhD Fellow in Health Psychology, developing research in the field of obesity in couples and health behavior change with the project “COuples’ OBesity (COOB): Conceptualization, measurement, development, and efficacy testing of an online-based couples’ intervention” (FCT Grant 2020.05357.BD). Her research interests include behavior change strategies, eHealth application for obesity interventions, mixed-methods (qualitative and quantitative methods), and psychometric validation of instruments. **Isabel Leal, **is a clinical and health psychologist and psychotherapist. PhD in Psychology from the Catholic University of Louvain, she has been a professor at ISPA – Instituto Universitário since 1983, where she is a full professor, researcher at the William James Center for Research (R&D) and currently dean. She developed professional activity within the Ministry of Health in the Civil Hospitals of Lisbon and in the Maternity Dr. Alfredo da Costa, where he founded and coordinated the Department of Clinical Psychology. She was Advisor to the National Commission to Fight AIDS, Consultant to the “Project to promote Mental Health in Pregnancy and early childhood” of the General Directorate of Health, President of the Portuguese Society of Health Psychology and President of the Jury of the Award Madalena Barbosa of the City Council of Lisbon, which promotes gender equality. Her research interests are currently focused on Health and Psychology’s interface in, particularly on sexual and reproductive health and gender issues and on the adjustment processes at transitions in the life cycle (adolescence, menopause, aging, disease, etc.). She has published more than 250 articles in specialized journals, 58 book chapters, 40 published books, and interacted with 218 collaborators in co-authorships of scientific papers.
